# High-resolution MRI of basilar atherosclerosis: three-dimensional acquisition and FLAIR sequences

**DOI:** 10.1002/brb3.103

**Published:** 2012-11-05

**Authors:** Tanya N Turan, Zoran Rumboldt, Truman R Brown

**Affiliations:** 1 Department of Neurosciences, Medical University of South CarolinaCharleston, South Carolina; 2 Department of Radiology, Medical University of South CarolinaCharleston, South Carolina

**Keywords:** Basilar artery, cerebral infarction, intracranial atherosclerosis, magnetic resonance imaging, plaque, pontine stroke

## Abstract

This case report describes the use of high-resolution magnetic resonance imaging (HRMRI) to visualize basilar artery atherosclerotic plaque in a patient with a pontine stroke. HRMRI with three-dimensional image acquisition was used to visualize plaque in several planes to localize arterial wall pathology. Fluid attenuated inversion recovery (FLAIR) sequences of the basilar artery showed wall thickening throughout the basilar artery wall and good contrast between the artery wall and cerebrospinal fluid.

## Introduction

Intracranial atherosclerosis (ICAD) is a common cause of stroke, but the pathology is not well understood because it cannot be easily studied in living patients. In extracranial carotid arteries, high-resolution magnetic resonance imaging (HRMRI) has shown good correlation with pathology (Underhill et al. 2010), but carotid HRMRI protocols cannot be directly applied to intracranial arteries because intracranial arteries are smaller, have unique histological features when compared with systemic arteries (Masuda et al. 2000; Levy et al. 2003), and are surrounded by cerebrospinal fluid (CSF) that may obscure the edges of the vessel wall on imaging (Qiao et al. 2011). In this report, we demonstrate that HRMRI using three-dimensional (3D) acquisition of T1-weighted, T2-weighted, and fluid attenuated inversion recovery (FLAIR) sequences allows visualization of atherosclerotic plaque in multiple planes and good contrast between the artery wall-lumen boundaries and artery wall-CSF boundaries.

## Case Report

A 55-year-old man with atherosclerotic risk factors presented with acute onset dysarthria and left hemiplegia consistent with the classic clinical syndrome pure motor hemiplegia due to basilar artery branch occlusion (Caplan 1989). He was found to have a paramedian pontine ischemic stroke seen on MRI. His stroke work-up revealed a basilar stenosis on computed tomography (CT) angiography, but was otherwise unremarkable. The patient underwent an institutional review board (IRB)-approved research HRMRI study on a Siemens 3T Trio scanner with 32-channel head coil. Sequences included 3D time-of-flight (TOF) magnetic resonance angiography (MRA) and single slab 3D acquisitions of the basilar artery including: T1-weighted pre- and postcontrast images (TR/TE 458/16, matrix 320 × 320, 11 slices, thickness 1.2 mm, field of view [FOV] 128 mm, flip angle [FA] 180°); T2-weighted images (TR/TE 1500/66, matrix 256 × 256, 11 slices, thickness 1.2 mm, FOV 104 mm, FA 180°, with fat suppression); and FLAIR images (TR/TE 2500/14, matrix 256 × 197, 11 slices, thickness 1.2 mm, FOV 100 mm, FA 90°, preparation pulse 140°). A normal volunteer with no basilar stenosis had a similar FLAIR sequence (all parameters were the same except FA 180°).

## Discussion

**Figure 1:**
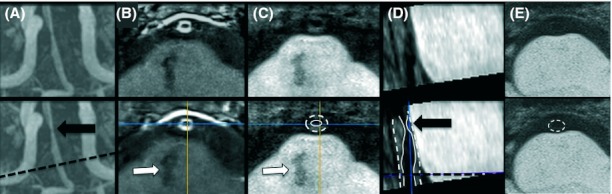
HRMRI of basilar atherosclerosis (top row unmarked and bottom row marked). (A) MRA of stenosis (black arrow) and nonstenotic artery at level of the pontine infarct (black dashed line); (B) enhancement of artery at level of infarct (white arrow); (C) FLAIR sequence with arterial wall thickening at level of the infarct on axial; and (D) sagittal views (outer wall [dashed white line] and lumen [thin white lines]) with focal stenosis above the level of infarct; (E) FLAIR images from a normal basilar artery with barely visible wall.

In this report, we demonstrate that HRMRI with 3D image acquisition can visualize intracranial plaque in several planes with good spatial resolution. Small improvements in spatial resolution are important when imaging small structures like the basilar artery. We found that an available FLAIR sequence with resolution of 0.4 mm showed good visualization of the atherosclerotic wall, likely due to the suppression of CSF signal. Unlike in T1/PD-weighted VISTA images wherein the vessel wall remained visible in normal volunteers (Qiao et al. 2011), in our FLAIR images, a normal artery wall is essentially imperceptible (Fig. 1E), suggesting that FLAIR may be sensitive for detection of vascular wall pathology.

The patient's pontine infarct was proximal to his basilar stenosis (Fig. 1A–D) and therefore not directly related to the basilar stenosis. However, at the level of the infarct, the artery wall enhanced consistent with neovascularization or “vulnerable” plaque (Kawahara et al. 2007) and on FLAIR, the artery wall was thickened. The sagittal FLAIR images reconstructed from the 3D acquisition showed wall thickening was variable. These characteristics suggest that atherosclerotic plaque may have overgrown the ostia of the perforating artery supplying the infarcted territory. Such instances of HR MRI identified nonstenotic plaque (defined as no measurable stenosis on MRA) have been associated with stroke due to occlusion of penetrating arteries (Klein et al. 2005, 2010; Li et al. 2009), similar to this case.

**Figure 2:**
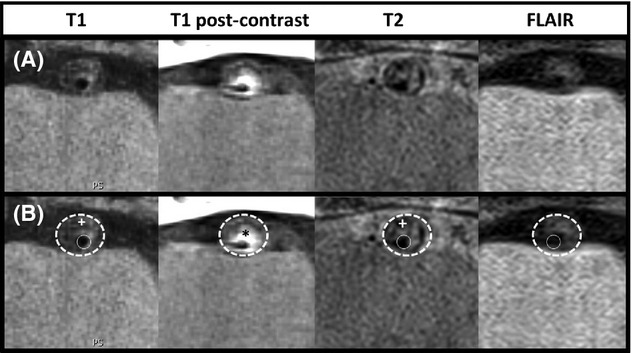
HRMRI of basilar atherosclerosis at level of the stenosis. Top row (A) T1 pre- and postcontrast, T2, and FLAIR images. Bottom row (B) shows same images with white dashed circle outlining artery and thin white circle outlining lumen. Lipid (white +) is isointense on T1 and hypointense on T2. Contrast enhancement of plaque (*) demonstrates ruptured fibrous cap.

At the stenosis (Fig. 2), the arterial wall was thickened on FLAIR with corresponding regions that were isointense on T1 and hypointense on T2, consistent with lipid in extracranial carotid HRMRI (Trivedi et al. 2004). The area adjacent to the artery lumen enhanced consistent with ruptured fibrous cap in extracranial carotid HRMRI (Wasserman et al. 2002). These findings demonstrate that plaque components (lipid core and fibrous cap rupture) may be visualized on HRMRI in ICAD. However, correlation between the HRMRI features and pathological specimens in ICAD has not yet been demonstrated. In addition, studies to determine the reliability of HRMRI for detecting high-risk plaque features and the prevalence of these features in ICAD are needed before their prognostic value can be determined.

## Conclusion

HRMRI with 3D image acquisition can visualize basilar artery plaque in multiple planes, allowing identification of plaque features that may contribute to the clinical presentation. The addition of FLAIR sequences helps localize arterial wall pathology by suppressing the surrounding CSF signal.
